# Free Radical Scavenging Capacities and Alleviating Actions of Polysaccharides Extract of *Termitomyces le-testui* on Methylprednisolone-Induced Immunodepression in Rats

**DOI:** 10.1155/2021/5893210

**Published:** 2021-11-08

**Authors:** Foncham Evans Ngwenah, Kada Sanda Antoine, Salah Martin, Tume Christopher, Oumar Mahamat

**Affiliations:** ^1^Laboratory of Biochemistry, Department of Biochemistry, Faculty of Science, University of Bamenda, Bamenda, Cameroon; ^2^Laboratory of Biological Sciences, Department of Biological Sciences, Faculty of Science, University of Bamenda, Bamenda, Cameroon

## Abstract

**Background:**

Natural products have been said to show immunomodulatory and antioxidant activities. The research study was aimed to assess the immunomodulatory and free radical scavenging activities of crude polysaccharide from dry mushroom fruiting bodies of *Termitomyces le-testui*.

**Materials and Methods:**

Hot water extract of polysaccharide extract of *T. le-testui* was prepared and tested in white albino Wister rats for its immunomodulatory activities effect on methylprednisolone-immunosuppressed animals. In addition, the radical scavenging activity of the polysaccharide was evaluated using nitrite and hydrogen peroxide.

**Results:**

The result of the study showed that the polysaccharide *T. le-testui* increases the phagocytic index, energy metabolism of macrophages, spleen index, and nitric oxide in a concentration-dependent manner in immunosuppressed animals. Also, it was observed that the extract increased dose-dependent total oxidative stress and thymus index. Finally, the crude polysaccharide-rich extract showed nitrite and hydrogen peroxide scavenging activity in a concentration-dependent manner.

**Conclusion:**

Polysaccharide-rich extract possesses immunomodulatory and antioxidant properties.

## 1. Introduction

Natural products, especially plants, are one of the immunomodulators that can provide an alternative to conventional chemotherapy for a large variety of diseases. As immunomodulators, they are especially important when the host defense mechanism has to be stimulated in cases of the impaired immune response. Some of them are important when a selective immunosuppression is desired. Mushrooms have been utilized in folk medicine since ancient times [1], but this concept gained little more credibility in the last decades as they exhibit significant therapeutic effect including antioxidant and immunomodulatory activities. This is partly due to a large number of substances with potential immunomodulatory activities that mushrooms possess [2, 3].

Mushrooms, as immunomodulators, they can suppress or enhance the immune system activities [4], and this offers them a large use to treat infections [5]. Among derived mushroom substances, there is ample evidence that polysaccharides exhibit various bioactivities such as antitumor, anticancer, antiviral, antibacterial, antifungal, anticoagulant, and immunological activities [6, 7]. Lentinan from *L. edodes* and *G. lucidum* polysaccharide, for example, through their immunostimulatory activities, showed important roles in maintaining tissue homeostasis and fighting diseases [8, 9]. In addition to the immunomodulation, polysaccharides also play an important role in the control of oxidative stress [10–12], thus contributing to the improvement of the immune response.

*T. le-testui* is an edible mushroom commonly consumed in Cameroon. The dry matter of *T. le-testui* revealed that it is made up of 59.02% of total carbohydrates, 23.6% of total sugars, and 0.84% of reducing sugars [13]. Data from various studies reported the immunomodulatory activities of *T. le-testui* [3, 14]. However, there is no data available on the active constituents of *T. le-testui.* Polysaccharides may be the bioactive constituents, but this remains to be demonstrated. The present study was therefore undertaken to assess the immune-modulatory activities against methylprednisolone-induced immunosuppression in rats and *in vitro* antiradical activities of polysaccharides extract of *T. le-testui.*

## 2. Materials and Methods

### 2.1. Materials

#### 2.1.1. Mushroom Specimen

**Figure 1:**
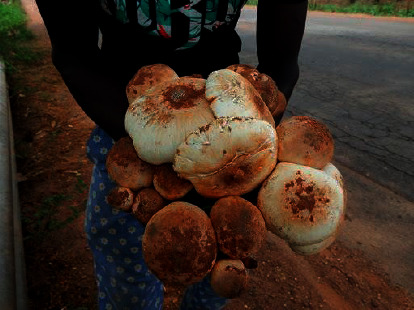
Photography of *T. le-testui*.

Fresh head bodies of *Termitomyces le-testui* (Figure 1) were obtained in the local market of Mbouda, in the West Region of Cameroon. Mushroom samples were authenticated by Dr. Njuonkou Andre Ledoux, a botanist in the Department of Biological Sciences, Faculty of Sciences, of the University of Bamenda, Cameroon. The obtained mushrooms were washed with tap water and cut into small pieces.

#### 2.1.2. Preparation Polysaccharides

Polysaccharides were extracted using hot water [15]. Fresh mushrooms were fully immersed in distilled water and incubated at 95°C in a water bath for six hours. The preparation was filtered and the filtrate was used to obtain the polysaccharides extract. The filtrate was mixed with ethanol (70%) at equal volumes and kept overnight at 4°C to precipitate the polysaccharides. After precipitation, the mixtures were then centrifuged at 1800 rpm during 10 min at 4°C temperature, and the pellets were then collected and dried at 30°C. The dried products representing the polysaccharide extracts were kept at about 4°C.

#### 2.1.3. Determination of Total Carbohydrate Content

The carbohydrate contents were determined by the slightly modified phenol-sulfuric acid method according to Masuko et al. [16]. Fifty microliters of crude polysaccharide solution was mixed with 150 *μ*L of concentrated sulfuric acid and immediately with 30 *μ*L of 5% phenol and then the mixture was kept at 90°C for 5 min. The absorbance of the mixture after cooling to room temperature was measured at 490 nm. The total carbohydrate content was calculated using a standard curve of D-glucose and it was found to be 75% of the dried extract.

### 2.2. In Vitro Evaluation of Antioxidant Activities

#### 2.2.1. Scavenging Activity Determination

The antioxidant activities of polysaccharides extract of *T. le-testui* were analyzed by assessing the free radical scavenging activities. The absorbance values measured in a UV-Vis spectrophotometer were used to determine the percentage inhibition of free radical scavenging activity by using the standard formula (F_1_) as reported by Bahatti et al. [17]:

(1) $$\begin{array}{c} \left(F_{1}\right)\cdot \%\text{ inhibition}=\left[\frac{A_{518}\left(\text{control}\right)-A_{518}\left(\text{sample}\right)}{A_{518}\left(\text{control}\right)}\right]\times 100, \end{array}$$

where *A*_518__(control)_ is the absorbance of control and *A*_518__(sample)_ is the absorbance of free radical + sample extract/standard.

#### 2.2.2. Nitrite-Scavenging Activity

Nitrite-scavenging activity was evaluated based on the absorbance at 520 nm using a UV-spectrophotometer according to the method reported by Kato et al. [18]. One milliliter of 1 mmol/L NaNO_2_ (Sigma Co.) solution was added to 1 mL of each sample, and the resulting mixtures were adjusted to a pH of 2.5 using 0.1 N HCl and 0.2 N citric acid solutions. Each sample was allowed to react at 37°C for 1 hour, after which 1 mL of each sample was taken from the solution and mixed thoroughly with 3 mL of 2% acetic acid and 0.4 mL of the Griess reagent. The solutions were stored at room temperature for 15 min. The Griess reagent was prepared by mixing an equal amount of 1% sulfanilic acid (Sigma Co.) and 1% naphthylamine (Sigma Co.), which were made with 3% acetic acid.

#### 2.2.3. Hydrogen Peroxide Scavenging Activity

The ability of the mushroom extracts to scavenge hydrogen peroxide was assessed according to Ruch et al. [19]. Mushroom extracts were added to 0.6 mL H_2_O_2_ solution (40 mM H_2_O_2_ was prepared with phosphate buffer, 0.1 M, pH 7.4) and the total volume was made up to 3 mL and mixed properly. Then the absorbance was measured at 230 nm using a spectrophotometer. Phosphate buffer, without H_2_O_2_, was considered as blank solution.

#### 2.2.4. Assay of Inhibition of Free Radicals Using Thiobarbituric Acid (TBA) Method

The TBA method was used to represent the inhibition of the production of carbonyl compounds degraded from the peroxides at a later stage. TBA test is used to measure the second product of peroxide oxidation such as aldehyde and ketone. The assay was done according to Kikuzaki and Nakatani [20]. To 2.0 mL of the sample solution, 1.0 mL of 20% aqueous trichloroacetic acid (TCA) and 2.0 mL of aqueous thiobarbituric acid (TBA) solution were added. The mixture was placed in a boiling water bath for 10 min. After cooling, it was centrifuged at 3000 rpm for 20 min and the absorbance was measured at 532 nm and recorded.

### 2.3. In Vivo Immunological Tests

#### 2.3.1. Animals

Albino rats (Wistar strain) aging 7 to 8 months and weighing 100–250 g were obtained from the laboratory of physiology and pharmacology of the University of Dschang. The animals were housed in cages under controlled conditions of temperature and alternating 12 hours cycle of light and darkness. They were given free access to standard rat pellet food and tap water *ad libitum*. All animals were acclimatized to the working environment 1 week before the beginning of the experiment. The experimental protocol was approved by the Ethics Committee of the Department of Biological Sciences of the University of Bamenda.

#### 2.3.2. Grouping and Treatment of Animals

After 1 week of acclimatization, rats were randomly distributed into 6 groups of 5 rats each. A group of 5 rats did not receive the immunosuppressive drug (methylprednisolone) (Group 1 or normal control). The remaining 5 groups of rats were all immunosuppressed by the injection of methylprednisolone via the tail vein (1 mL/kg of animal weight) before receiving the other treatments. One group of the immunosuppressed rats (Group 2 or negative control) received distilled water. Another group (Group 3 or positive control) received intraperitoneal injection of BCG (2.10^3^ CFU/mL) for the entire treatment period. Finally, the remaining immunosuppressed groups of rats (Groups 4, 5, and 6) were orally treated by gavage with the different doses of the mushroom extract 25, 50, and 100 mg/kg, respectively. Treatment was done every day for 10 days.

#### 2.3.3. Sample Collection

For this part of the study, two animal groupings were done. The first grouping was to evaluate the phagocytic index and lymphoid organ index, and the second grouping was to evaluate the energy metabolism in intraperitoneal macrophages, production of nitric oxide, and total oxygen radical. The treatment was done for 10 days. Thereafter, animals were administered a mixture of ketamine (dose) and Diazepam (dose) (ratio 1 : 3) and blood was collected by cardiac puncture in dry essay tubes to assess the serum nitric oxide and total oxygen radical or through the orbital venous to evaluate the phagocytic index. Peritoneal macrophages were obtained by washing the peritoneal cavity in the phosphate buffer. Spleen and thymus were collected and kept in formalin solution 10% for histological analysis.

#### 2.3.4. Assessment of Phagocytic Index

Phagocytic index was assessed through the carbon clearance test as described by Honghui et al. [21] with some modification. Thirty rats treated as described previously were intravenously injected with diluted India ink at 100 *μ*L/10 g body weight. Blood specimens were collected at 2 min (*t*_1_) and 10 min (*t*_2_) from the retinal venous plexuses and 20 *μ*L of the collected blood was then mixed with 2 mL 0.1% Na2CO3. The absorbance at 600 nm was measured on a UV-visible spectrophotometer with 0.1% Na2CO3 as the blank. The liver and the spleen were weighed, and the phagocytic index was calculated as follows:

  (*F*_2_): *K*= (lg OD_1_ − lg OD_2_)/(*t*_2_ − *t*_1_), where OD_1_ was optical density for *t*_1_ and OD_2_ was optical density for *t*_2_
  (*F*_3_): phagocytic index =  $\sqrt[{3}]{k}\times A/\left(B+C\right)$, where A is the body weight, B the liver weight, and C the spleen weight

#### 2.3.5. Evaluation of Energy Metabolism in Peritoneal Macrophages

Peritoneal macrophages were harvested by washing the peritoneum in 3 mL of phosphate buffer saline (PBS). Peritoneal cells were immediately counted using a hemocytometer (Malassez cell). A cell suspension (density; 1 × 10^3^ cells/mL) was prepared and 2 mL of this suspension was mixed with 1 mL of MTT (3-(4,5-dimethylthiazol-2-yl)-2,5-diphenyl tetrazolium) solution (1% in PBS). After 2 hours of incubation, 1 mL of 1N HCl was added and the mixture was allowed to stand for 15 min after which the optical density was read at 540 nm. The mean of the optical density in treated animal was compared to that of control to evaluate the energy metabolism of the cells (reference).

#### 2.3.6. Evaluation of Radical Compounds Production

The crude polysaccharides of *T. le-testui* were analyzed for their effect on radical compounds production. This test is based on iron catalyzed breakdown of hydroperoxides into alkoxyl (RO۰) and peroxyl (ROO۰) radicals which react with the chromogen (N, N-dimethyl -*p*- phenylenediamine sulphate) towards formation of a colored compound, the absorbance of which is photometrically detectable. The intensity of the color correlates directly with the quantity of radical compounds, according to Lambert–Beer's law, and it can be related to the oxidative status of the sample. This was done by the method described by Pinaki et al. [22]. The method makes use of chromogen reagent solution (23.5 mg of N,N-dimethyl- p-phenylenediamine sulphate (Aldrich, Sigma) in 10 mL of 20 millimolar PBS (pH 7.4)) and kept in 4–8°C. Briefly, 100 microliters of plasma diluted to 20 times in PBS was dissolved in 1 mL of acetate buffer. 25 microliters of working chromogen solution was added, and absorbance was taken at 505 nm by 6 min time-scan in UV-Vis spectrophotometer. The absorbance value was obtained for each sample against blank and compared to the curve obtained using H_2_O_2_.

#### 2.3.7. Evaluation of Nitric Oxide Production

The crude polysaccharides of *T. le-testui* were analyzed for their effect on nitric oxide production as described by Pinaki et al. [22]. Griess reagent (made by mixing an equal amount of 1% sulfanilic acid and 1% naphthylamine which was made with 3% acetic acid) is the principal reagent used. The collected serum sample was made up to 1 mL using 20% phosphate buffer. To each sample of serum, 1 mL of the working reagent (Griess reagent) was added and allowed to stand for 5 min and absorbance was measured at 540 nm. This was done in duplicate for each sample, and the absorbance was recorded. A standard curve was produced using NaNO_2_ as standard solution.

#### 2.3.8. Histological Analysis of Spleen and Thymus

Spleen and thymus of sacrificed animals were collected, washed in cold phosphate buffer saline, and blotted dried. They were weighted to obtain the absolute organ weight. The organ index was calculated as follows:

(2) $$\begin{array}{c} \left(F_{4}\right)\cdot \text{organ index}\left(\frac{\text{mg}}{\text{g}}\right)=\frac{\left(\text{weight of thymus or spleen}\right)}{\text{body weight}}. \end{array}$$

Organs were immediately fixed in 10% formalin until the day of histological analysis. Later, the ﬁxed tissues were dehydrated and then the samples were cleared in 2 changes of xylene. Samples were then impregnated and embedded in paraffin, then embedded, and blocked out. Sections of 4–6 *μ*m thickness were cut using a rotary microtome and mounted on glass slides. After staining with Hematoxylin and Eosin (H&E), the sections were imaged using the Pannoramic Viewer (3DHISTECH, Budapest, Hungary) connected to a microscope.

### 2.4. Statistical Analysis

Experimental data obtained were expressed as the mean ± standard deviation of the experiment done in triplicate. Data was analyzed using one-way analysis of variance, followed by post-Students tests. Values of *p* < 0.05 were considered as indicative of significance. All calculations were carried out using the GraphPad Prism® V5.03 software (GraphPad Software Inc.®, CA, USA).

## 3. Results

### 3.1. Nitrite and Hydrogen Peroxide Scavenging Activity of Crude Polysaccharides of *T. le-testui*

The polysaccharide-rich extract of *T le-testui* was tested for its antiradical activity by evaluating its effect on nitrite and hydrogen peroxide scavenging activity (Table 1). The result showed that a concentration-dependent increase of the inhibition percentage of free radical activity was observed with extract. This activity of polysaccharides was similar to the effect of vitamin C (100 mg) at 11.06 mg/mL polysaccharides extract while 8.3 mg/mL was significantly different from the effect of vitamin C.

### 3.2. Free Radicals Scavenging Using Thiobarbituric Acid (TBA) Method

**Figure 2:**
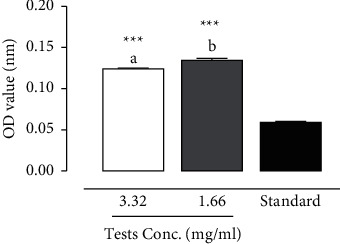
Antioxidant activity of polysaccharides extract of *T. le-testui* as measured by the TBA method at 532 nm and compared to standards (vitamin C).

The TBA method was used to evaluate the inhibition of the production of carbonyl compounds degraded from the peroxides at a later stage. The results showed that the polysaccharides active extracts of *T. le-testui* caused a decrease in the production of carbonyl compounds in a concentration-dependent manner (Figure 2). The production of carbonyl compounds was significantly less with the polysaccharides extract than the synthetic antioxidant vitamin C.

### 3.3. Effect of *T. le-testui* Crude Polysaccharide on Phagocytic Index and Peritoneal Cells Metabolism in Methylprednisolone-Immunosuppressed Rats

*In vivo* administration of methylprednisolone resulted in significant decreases (*p* < 0.05) in the phagocytic index and energy metabolism of peritoneal macrophages (Table 2). However, BCG as well as polysaccharides extract of *T. le-testui* treatments prevented the increase and maintained these parameters near the normal level and even above normal for the dose of extract of 100 mg/kg. Polysaccharides extract treatments were also able to reverse the decreases in phagocytic index caused by methylprednisolone to near normal. No significant differences were observed with BCG treatment.

### 3.4. Effect of Crude Polysaccharide Extract of *T. le-testui* on Organ of Immune System

The treatment with methylprednisolone significantly decreased the spleen and thymus weight (Table 3). Administration of polysaccharides extract of *T. le-testui* significantly caused the increase of spleen index and thymus index at the doses of 100 mg/kg and 50 mg/kg, respectively. The reversal effect of the extract brought back the organs to normal. There was no difference compared to the effect of BCG.

### 3.5. Serum Level of Nitric Oxide and Radical Compounds of Methylprednisolone-Immunosuppressed Rats Treated with the Crude Polysaccharide of *T. le-testui*

Administration of methylprednisolone to rats significantly decreased the serum NO and radical compounds level compared to normal animal. In concentration-dependent manner, treatment with polysaccharides extracts of *T. le-testui* resulted in significant increase in the levels of NO (Table 4). The effect of the treatment with polysaccharides extract was similar to that of BCG. Moreover, polysaccharides extract of *T. le-testui* reversed the effect of methylprednisolone with the highest effect observed with the lowest dosage.

### 3.6. Effects of Polysaccharides-Enriched Extracts of *T. le-testui* on Lymphoid Cells Accumulation in Spleen and Thymus Tissues

**Figure 3:**
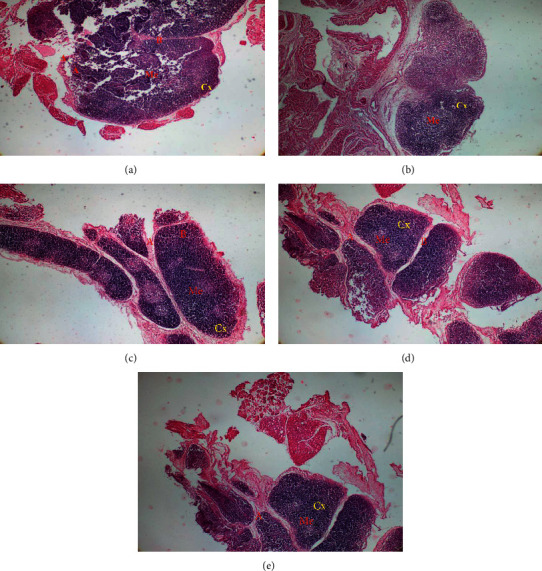
Microarchitecture of rat thymus of different groups (control and extract-treated groups). Normal group: untreated animals; negative: animals receiving methylprednisolone only. (a) Cross section in a rat thymus of normal group showing a lobule of the thymus with dense cortex (Cx), medulla (Me), interlobular (A), and intralobular (B) (H&E, X 100). (b) Cross section in a rat thymus of negative group showing the lobule of the thymus with less dense cortex (Cx) and the medulla (Me), interlobular (A), and intralobular (B) (H&E, X 100). (c) Cross section in a rat thymus of 25 mg/kg-treated group showing the lobule of the thymus with dense cortex (Cx) and the medulla (Me), interlobular (A), and intralobular (B) (H&E, X 100). (d) Cross section in a rat thymus of 50 mg/kg-treated group showing the lobule of the thymus with dense cortex (Cx) and the medulla (Me), interlobular (A), and intralobular (B) (H&E, X 100). (e) Cross section in a rat thymus of 100 mg/kg-treated group showing the lobule of the thymus with very dense cortex (Cx) and the medulla (Me), interlobular (A), and intralobular (B). (H&E, X 100).

**Figure 4:**
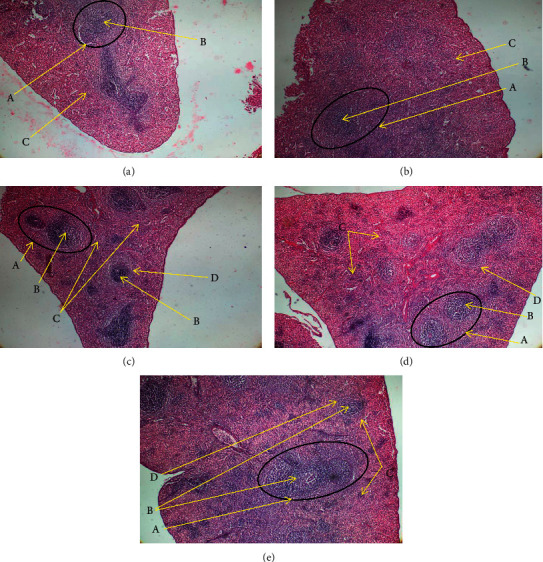
Microarchitecture of rat spleen of different groups (control and extract-treated groups). Normal group: untreated animals; negative: animals receiving methylprednisolone only. (a) Cross section of a rat spleen of control group showing the white pulp of the spleen with germinal center (B) and the red pulp (C) (H&E, X 100). (b) Cross section of a rat spleen of negative group showing white pulp (A) with poor germinal center (B) and the red pulp (C) (H&E, X 100). (c) Cross section of a rat spleen of 25 mg group showing white pulp (A) with very dense germinal center (B), marginal zone (D), and the red pulp (C) (H&E, X 100). (d) Cross section of a rat spleen of 50 mg group showing white pulp (A) with dense germinal center (B), marginal zone (D), and the red pulp (C) (H&E, X 100). (e) Cross section of a rat spleen of 50 mg group showing white pulp (A) with dense germinal center (B), marginal zone (D), and the red pulp (C) (H&E, X 100).

Methylprednisolone resulted in significant reduction in richness of lymphocytes in spleen and thymus of rats. In thymus, polysaccharides extract of *T. le-testui* treatment also increased the richness of lymphocytes (thymocytes) as compared to that of animals not treated (negative control). This was demonstrated by the dense thymus cortex observed in treated animals (Figure 3). The administration of polysaccharides extract of *T. le-testui* also affected the lymphoproliferative activity of spleen. The treatment has caused the white pulp to have a dense germinal center compared to the negative control (Figure 4).

## 4. Discussion

Since a long time ago, mushrooms have been known for numerous pharmacological activities. This also includes immunomodulatory and antioxidant activities [23]. In the present study, we assessed the reversal effect of crude polysaccharide extract on methylprednisolone-immunosuppressed effect in white albino Wistar rats and its antioxidant activities. Nowadays, there is considerable effort to identify natural substances that can protect against oxidative stress or stabilize or deactivate free radicals in an immunosuppressed state. Nitric oxide radical is one of the potent radicals that acts by generating full name of NO_2_ (NO_2_) [24]. The polysaccharide extract of *T. le-testui* was found to decrease the amount of radical. Moreover, the polysaccharides extract of *T. le-testui* has demonstrated a potential to have a H_2_O_2_-scavenging activity demonstrating that *T. le-testui* has potent antioxidant properties. Hydrogen peroxide (H_2_O_2_), a biologically relevant, nonradical oxidizing species, can be formed in tissues through oxidative processes. H_2_O_2_ produced may result in the production of hydroxyl radicals (•OH) causing the lipid peroxidation [25] or alteration of the structure and function of many cellular components [26]. Thus, the polysaccharide-rich extract of *T. le-testui* may counteract the oxidative effect of H_2_O_2_. Further demonstration of the radical scavenging activity of *T. le-testui* was observed in TBA test assay. Though the scavenging activity of polysaccharide-rich extract of *T. le-testui* noticed in TBA assay test was lower compared to that of vitamin C, this result supports its radical scavenging potential. Together, the results of this study demonstrated the free radicals scavenging power of the polysaccharides extract of *T. le-testui,* as it has been reported for numerous natural products [27].

Several studies reported the immunomodulatory effects of the extract of *Termitomyces* species through a remarkable improvement of the antibodies production, and also the phagocytic activities [3, 14]. In this study, the crude polysaccharides of *T. le-testui* were found to reverse the immunosuppressed effect of methylprednisolone. Firstly, this was demonstrated by increasing the phagocytic index towards the normal level in a dose-dependent manner. Secondly, the polysaccharides extract of *T. le-testui* may enhance the metabolism rate of peritoneal macrophages from the methylprednisolone-treated rats. Macrophages have a strong engulf particulate matter function *in vivo* or *in vitro* [28], and macrophage metabolism has been also reported as a crucial indicator for the immune function [29]. This activity of macrophages is affected by the methylprednisolone [30, 31]. Thus, the results of this study indicate the potential immunostimulatory effect of *T. le-testui*, through which it may stimulate the immune cells and improve the body's immune function [32, 33]. Methylprednisolone is also known to cause lymphocytic depletion in spleen in rats [34]. The polysaccharide-rich extract of *T. le-testui*, furthermore, demonstrated that it can improve the thymus and spleen indices in rats treated with this immunosuppressive drug. This is evidence that *T. le-testui* improves the strength of the body's innate immune function [35–37]. This effect of *T. le-testui* is also supported by the lymphocytes colonization of thymus and spleen as demonstrated by the histological analysis of these organs reported for other mushrooms species such as *Lentinula edodes* [38]. As a result of phagocytes activation, reactive oxygen species and nitrogen reactive species are generally produced to participate in the intracellular killing of pathogens and mediate inflammatory reaction [39]. The results of this study also showed that polysaccharide-rich extract of *T. le-testui* has a potential to increase the NO and total radical compounds in methylprednisolone-treated rats. Once more, this demonstrated that polysaccharides of *T. le-testui* can boost the body's immune system and can reverse the symptoms of low immunity.

In conclusion, the polysaccharide-rich extract showed potent antioxidants and immunomodulatory effects on different components of the innate and adaptive immune response. These pharmacological activities can justify the traditional use of this mushroom for the treatments of several diseases in relation to the immune response.

## Figures and Tables

**Table 1:** Radical scavenging activity of crude polysaccharides of *T. le-testui* as measured by the nitrite and H_2_O_2_-scavenging tests and compared to standards (vitamin C).

Assays	Nitrite-scavenging activity		H_2_O_2_-scavenging activity	
	OD	%	OD	%
Extract (mg/mL)				
8.3	1.33 ± 0.04	9.05 ± 3.27^*∗*^	0.59 ± 0.06	12.07 ± 0.58^*∗*^
11.06 mg/mL	0.76 ± 0.05	48.15 ± 3.47	0.54 ± 0.001	19.50 ± 9.61

Vitamin C (mg/mL)				
100	0.77 ± 0.01	47.26 ± 1.14	0.52 ± 0.004	21.16 ± 0.96

Asterisks (^*∗*^*p* < 0.05) indicate the significant difference as compared to the positive control (vitamin C).

**Table 2:** Effect of *T. le-testui* crude polysaccharides on phagocytosis and energy metabolism in methylprednisolone-treated rats.

Treatments	Dosage	Phagocytic index	Energy metabolism
Nor. control	—	12.56 ± 0.64^*∗∗*^	0.11 ± 0.028^*∗*^
Neg. control	1 ml	5.31 ± 0.49	0.07 ± 0.007
BCG (2.10^3^ CFU/mL)	200 *µ*l	10.11 ± 1.81^*∗*^	0.11 ± 0.014^*∗*^
Extract (mg/kg)	25	13.84 ± 5.82	0.09 ± 0.082
	50	13.99 ± 4.14^*∗*^	0.21 ± 0.071^*∗*^
	100	18.73 ± 4.73^*∗∗*^	0.15 ± 0.079^*∗*^

Neg. control: prednisolone-treated animal and receiving the vehicle (saline solution) and Nor. control: normal control. Asterisks (^*∗*^*p* < 0.05, ^*∗∗*^*p* < 0.01, and ^*∗∗∗*^*p* < 0.001) indicate the significant difference as compared to negative control.

**Table 3:** The effect of crude polysaccharides extract of *Termitomyces le-testui* on spleen and thymus index.

Treatment		Spleen index	Thymus index
Neg. control	1 ml H_2_O	0.0032 ± 0.0008	0.00019 ± 0.00001
Nor. control	—	0.0076 ± 0.0005^*∗*^	0.00041 ± 0.00003^*∗*^
BCG (2.10^3^ CFU/ml)	200 *µ*l	0.0043 ± 0.0017^*∗*^	0.00046 ± 0.00005^*∗*^
Extract (mg/kg)	25	0.0045 ± 0.0005	0.0010 ± 0.00051
	50	0.0052 ± 0.0001^*∗*^	0.0010 ± 0.00022^*∗*^
	100	0.0055 ± 0.0005^*∗*^	0.00081 ± 0.0003^*∗*^

Neg. control: prednisolone-treated animal and receiving the vehicle (saline solution) and Nor. control: normal control. Asterisks (^*∗*^*p* < 0.05, ^*∗∗*^*p* < 0.01, and ^*∗∗∗*^*p* < 0.001) indicate the significant difference as compared to negative control.

**Table 4:** Effect of the crude polysaccharide of *T. le-testui* on serum level of nitric oxide and reactive oxygen species production in methylprednisolone-treated rats.

Treatment		NO	ROS
Nor. control	—	1.340 ± 0.0028^*∗∗∗*^	297.40 ± 1.31^*∗∗∗*^
Neg. control	1 ml H_2_O	0.3151 ± 0.062	60.77 ± 10.46
BCG (2.10^3^ CFU/ml)	200 *µ*l	0.5557 ± 0.0070^*∗*^	125.7 ± 18.88^*∗∗∗*^
Extract (mg/kg)	25	0.7508 ± 0.376	235.2 ± 41.19^*∗∗∗*^
	50	1.016 ± 0.524^*∗*^	238.9 ± 103.39^*∗∗*^
	100	1.045 ± 0.343^*∗∗*^	218.7 ± 69.20^*∗∗*^

Neg. control: prednisolone-treated animal and receiving the vehicle (saline solution) and Nor. control: normal control. Asterisks (^*∗*^*p* < 0.05, ^*∗∗*^*p* < 0.01, and ^*∗∗∗*^*p* < 0.001) indicate the significant difference as compared to negative control.

## Data Availability

Data will be available under request to the corresponding author.
